# Barriers to implementing advanced skills in trauma and emergency nurse specialist practice

**DOI:** 10.4102/hsag.v30i0.2951

**Published:** 2025-07-11

**Authors:** Haroldene Stevens, Cornelle Young, Tshepo L. Motsepe, Tendani Mabuda

**Affiliations:** 1 Department of Health and Wellness, Western Cape College of Nursing, South Cape Karoo Nursing Campus, George, South Africa; 2 Department of Nursing and Midwifery, Faculty of Medicine and Health Sciences, Stellenbosch University, Cape Town, South Africa

**Keywords:** trauma and emergency nursing, advanced skills, barriers, scope of practice, skills implementation, nurse specialists

## Abstract

**Background:**

A postgraduate specialist programme in trauma and emergency nursing is offered to help address the high burden of injuries and emergencies in the South African healthcare system. The trauma and emergency nurse specialists’ advance skills and expertise have been associated with improved patient outcomes, yet they continue to face barriers that constrain the full scope of their practice.

**Aim:**

This study aimed to explore and describe the perceptions of trauma and emergency nurse specialists on barriers to skill implementation.

**Setting:**

The study was conducted in the trauma and emergency departments of a tertiary hospital providing Level I services and a district hospital providing Level II services at two public hospitals in the Western Cape province, South Africa.

**Methods:**

An exploratory-descriptive qualitative design with purposive maximum variation and snowball sampling was utilised. Semi-structured interviews were conducted with 10 specialist nurses among a population of 33 with a post-registration qualification in Medical and Surgical Nursing Science: Trauma and Emergency. Thematic analysis of data was carried out and adherence to the criteria of dependability, credibility, transferability and confirmability ensured trustworthiness in the study.

**Results:**

Three themes emerged, which include uncertainty regarding legal accountability and specialised practice responsibility, oppressive practice environments not conducive to skill implementation and the disregard for trauma and emergency nurse specialists’ role and value of specific skills.

**Conclusion:**

Job descriptions and scope of practice (SOP) are not adapted after specialist training in trauma and emergency nursing, resulting in a lack of recognition of specialised skills by the interdisciplinary team and management, uncertainty among these specialist nurses about what they are allowed to do with their new skills and a subsequent inability to practice in their full capacity in service of the population in need.

**Contribution:**

Improved utilisation of the specialised practice of these trauma and emergency nurse specialists within their expanded scope to cope with the high burden experienced in the emergency rooms of South Africa.

## Introduction

The global healthcare crisis demands nursing care at an advanced level, enabling task-sharing or shifting of the patient loads from overloaded physicians. Professional nurses with specialist care training are needed to manage the complex health needs of countries (Horvath et al. 2023:2), including the ‘colliding epidemics’ of human immunodeficiency virus (HIV) and tuberculosis, chronic illness and mental health, injury and violence and maternal, neonatal and child mortality (Achoki et al. 2022:1). Lecky et al. (2020:68) found that harnessing such interdisciplinary collaboration to improve emergency care in low- and middle-income countries (LMICs) was cost-effective, enhancing access to fast-growing populations with less healthcare coverage, improved patient outcomes and served as best practice in the emergency room. Hence, nurses educated at a higher level should utilise their further training to full capacity to support populations’ coverage and quality of healthcare delivery (World Health Organization [WHO] 2020:9). Full utilisation of trauma trained nurses will assist in reducing the mortality and disability rates of trauma patients, improve the quality of trauma nursing and rescue and alleviate the overcrowding in emergency departments (Liu et al. 2024:5).

Globally, barriers exist for advanced practice nurses (APN) to practise to the full extent of their education and scope of practice (SOP) because of problems with legislation, role ambiguity, autonomy and resistance to roles (Torrens et al. 2020:2). Kandrack, Barnes and Martsolf (2019:215) mention the limited APN practice in many states of the United States (US) because of such SOP regulations that impose restrictions on nurse practitioner practice and prescriptive authority to protect the quality of care. These authors also observe that no differences were found in the quality of care provided by APNs in those US states without these restrictions. Similar to the US, SOP regulations in South Africa by the South African Nursing Council (SANC 2022:1) govern the authority related to prescriptive and practice authority, which explains the extent to which professional nurses can independently diagnose, treat and prescribe to patients. In the South African LMIC context, regulations and resources are not very adaptable, especially in areas of service delivery, where physician availability is juxtaposed with the needs of the growing population (Department of Health 2015:1). In the Western Cape, South Africa, national budget constraints compelled the Provincial Department of Health (WCDoH) to improve staff productivity and efficiency to safeguard health service delivery and patient care (WCDoH 2019:162). In this austere climate, it is imperative that the competencies of the trauma and emergency nurse specialists are practised, as their qualification allows them to take on these more specialist duties to the benefit of the service.

Trauma and emergency healthcare demand often exceeds the resource capacity of the staff categories available, with resultant gaps in care, increasing patients’ lengths of stay and medical errors (Crawford 2019:1). Subsequently, factors that hinder skill utilisation for enhanced staff productivity and efficiency need to be investigated and addressed. The aim of this study was, therefore, to explore and describe the perceptions of trauma and emergency nurse specialists regarding the factors that hinder the implementation of their specialised skills in their practice within the public health sector of the Western Cape (Stevens 2021).

## Research methods and design

### Study design

A descriptive qualitative design allowed the researcher to obtain insight into the perspectives of trauma and emergency nurse specialists on the barriers to advanced skill implementation.

### Setting

The trauma and emergency units of two public hospitals in the Western Cape province of South Africa were selected. One tertiary hospital providing level I trauma and emergency services and a district hospital providing level II trauma and emergency services in the Cape Metropole were selected to obtain a variety of participants’ experiences at different units and levels of trauma and emergency care (Stevens 2021). The district hospital receives patients from clinics and day hospitals in its vicinity and refers patients to the tertiary hospital as needed for specialist care. The tertiary hospital is one of two in the Cape Metropole, which receives referrals from all the district and regional hospitals in the whole Western Cape province. Authors Zaidi et al. (2019:1) mention from their study about the burden of trauma at a district hospital in the Western Cape, that these level hospitals have inadequate emergency and trauma staff and are resource limited. Substance abuse and gang-related violence are the leading causes of emergency attendance, and these hospitals deal with intentional injuries such as homicide and gunshot wounds, apart from the regular clients of an emergency care centre (WCDoH 2019:4).

### Study population and sampling strategy

The study population comprised 33 permanently employed specialist nurses with a post-registration qualification in Medical and Surgical Nursing Science: Trauma and Emergency, with 24 employed in the tertiary hospital and nine in the district hospital. Participants were purposively selected using a combination of maximum variation sampling and snowball sampling to capture the widest range of perspectives possible considering gender, experience and proportion of participants in the two settings.

### Data collection

An interview guide with questions to meet the objectives of the study, and probes to elicit rich information from the participants was developed. A broad question about the utilisation of their specialised skills was asked: Please tell me everything about your experiences in terms of the implementation or utilisation of your specialised skills as a trauma and emergency nurse specialist in your trauma and emergency practice. This question was followed by questions about barriers to the application of such skills, as well as recommendations to support the appropriate use of their specialist skills. Face validity of the interview guide was provided by the study supervisors and colleagues involved in the research methodology. A pilot interview with a trauma and emergency nurse specialist from the tertiary hospital served to determine the validity of the interview guide to provide the necessary data. No changes were necessary to the interview guide, and the pilot interview was included in the final data.

Data were collected from October 2020 to December 2020 by the researcher, using semi-structured interviews during real-time virtual calling, as this method was preferable during the coronavirus disease 2019 (COVID-19) period. Interview calls were booked for 1 h, at a time convenient to participants, and actual interviews lasted between 40 min and an hour. Data saturation was achieved with the tenth interview providing repetition in information similar to the other interviews, and the finding that the obtained data were sufficient to meet the objectives of the study.

### Data analysis

Thematic analysis of data using the six phases proposed by Braun and Clarke (2020:331–333) helped to develop themes that captured the central ideas embedded in the data. Interview transcriptions were done and rechecked by the researcher, allowing for familiarisation and reflective notes on the meaning and patterns of comparable experiences detailed in the data. Subsequent manual coding allowed for the analysis of intricate data content with intuition and insight, bearing in mind the significance that participants attached to the content they shared (Stevens 2021). Elementary segments of codes were grouped into broader themes and reviewed by the researcher and a co-coder to form coherent entities that captured the essence of the complete data set. Contemplation of each theme allowed for theme naming in the specific context of answering the research question.

### Trustworthiness

Meticulous integration of validity techniques was applied during all steps of the research process, based on the criteria of dependability, credibility, transferability and confirmability for trustworthiness, as set out by Lincoln and Guba (1985:290). Observance of credibility was facilitated by adequate interview time for clarification and probing to appreciate the context of experiences and shared meanings. Moreover, considerable time was invested in making sense of the data, coding and the development of themes to provide a true reflection of the participants’ views. Member checking confirmed the accuracy of verbatim interview transcripts and direct verbatim quotes used to discuss and support the findings of the study. Co-coding by a second, independent coder confirmed coding reliability together with a transparent audit trail, which ensured the confirmability and dependability of the study findings. For transferability, the whole process taken was set out meticulously, so that interested parties could confirm the replicability of the study for their circumstances.

### Ethical considerations

Ethical clearance to conduct this study was obtained from the Stellenbosch University Health Research Ethics Committee (No. S19/10/277) and the Provincial health facility at Tygerberg Hospital (No. 40/2009). The researcher created a Google form link to procure consent and provide information about the study, which was sent to potential participants to indicate consent and understanding of study information by clicking on boxes or choosing from dropdown boxes. Automatically captured Google link responses were then reviewed to identify consenting participants who were subsequently contacted to arrange for interviews. Confidentiality and privacy of data were ensured by de-identifying the data and saving it on a password-protected computer to be discarded after five years.

## Results

Ten participants were interviewed, with six from the tertiary hospital and four from the district hospital. Three themes captured the perceived barriers to specialist skill implementation: Uncertainty regarding legal accountability and specialised practice responsibility, oppressive practice environments not conducive to skill implementation and disregard for trauma and emergency nurse specialists’ role and value of specific skills. Themes and sub-themes are presented in Table 1.

**TABLE 1:** Themes and sub-themes.

Themes	Sub-themes
1. Uncertainty regarding legal accountability and specialised practice responsibility	1.1 Perceived scope of practice limitations 1.2 Job description not aligned with specialised skills
2. Oppressive practice environments not conducive for skill implementation	2.1 Service demands overwhelmed capacity 2.2 Inappropriate staff allocation and skill mix 2.3 Limited practice opportunities lead to the loss of specialised skill competencies
3. Disregard for trauma and emergency nurse specialists’ role and value of specific skills	3.1 A lack of awareness, recognition and appreciation of specialised role and skill sets in the multidisciplinary team

*Source:* Adapted from Stevens, H., 2021, ‘Trauma and emergency nurse specialists’ perceptions of factors that hinder and facilitate the implementation of specialised skills within the public health sector in the Western Cape Province’, Thesis in fulfilment of Masters degree, Stellenbosch University, Cape Town

### Theme 1: Uncertainty regarding legal accountability and specialised practice responsibility

As per Table 1, uncertainty about legal accountability and specialised practice responsibility among trauma and emergency nurse specialists has the sub-themes of perceived SOP limitations, as well as job descriptions not aligned with specialist skills.

#### Perceived scope of practice limitations

The requirements of the institution determined that trauma and emergency nurse specialists had to wait for a doctor to initiate treatment. The legal responsibility, accountability and specialist practice of trauma and emergency nurse specialists are not recognised in SOPs, as these still only hold medical practitioners responsible for ordering emergency treatment, which delays timely intervention in acute medical emergencies:

‘An MI [*myocardial infarction*] patient for example, you know what signs to look for. You do the ECG, the interpretation of the ECG. Now, you must first wait for the doctor before you can give stat [*immediate*] treatment. Because it’s not in your Scope of Practice. You know you’re supposed to give Aspirin [*and*] Isordil. You know what treatment you’re supposed to give, but it’s not within your Scope of Practice [*to give that treatment without a doctor’s prescription*].’ (Participant 4, female, trauma and emergency nurse specialist)

The specialised skill of performing and interpreting an arterial blood gas (ABG) test that all trauma and emergency nurse specialists are competent in conducting is forbidden without a doctor’s prescription. Therefore, trauma and emergency nurse specialists often practice in the same capacity as a professional nurse without further specialisation:

‘We are not allowed to do ABG’s which is ridiculous. Because we know how to do it and we know on what patients to do the ABG on.’ (Participant 4, female, trauma and emergency nurse specialist)

‘… as a specialist or nurse specialist, my skills are not utilised completely. We basically do everything that a general nurse does, are capable of, and there isn’t much of the specialisation part that we play.’ (Participant 7, female, trauma and emergency nurse specialist)

Among their recognised competencies trauma and emergency nurse specialists are certified in Advanced Life Support (ALS). This enables them to apply a variety of life-saving treatments in settings where the time for definitive treatment plays a critical role in successful patient outcomes. The use of the life-saving skill in defibrillation (‘defib’) and cardioversion (‘cardiovert’) in a patient with a cardiac emergency is one such situation:

‘You know when to defib the patient, you know when to cardiovert, you know what signs to look out for, but yet, you can’t do it. You need to wait for the doctor, it is a waste of time, but you can’t just go ahead and do things out of your own.’ (Participant 4, female, trauma and emergency nurse specialist)

#### Job description not aligned with specialised skills

Participants reported that the specialised skills they had acquired upon completing the programme were not added to their job descriptions although they had demonstrated competence in certain specialised skills during their training:

‘My job description is still the same as … how many years ago as any registered RN [*professional nurse without specialised training*] job description. There’s nothing particular about trauma trained in that job They should look at the job description again and then add [*to it*] in such a way that it is for a skilled trauma trained person, and also considering her Scope of Practice.’ (Participant 2, female, trauma and emergency nurse specialist)

Scope of practice and job descriptions for specialist practice have not been adapted despite new certified competencies gained, creating frustration and uncertainty about their own responsibility versus that of medical practitioners. Specialised skills are thus not optimally utilised within trauma and emergency practice settings.

### Theme 2: Oppressive practice environments not conducive to skill implementation

The second theme that emerged from the data analysis, as illustrated in Table 1, pertains to oppressive practice environments that are not conducive to the implementation of specialised skills. Three sub-themes relate to this main theme, namely inappropriate staff allocation and skills mix, service demands that overwhelm capacity and limited ability to practice, leading to loss of specialised skills.

#### Inappropriate staff allocation and skill mix

Staff allocation patterns limit practice opportunities for trauma and emergency nurse specialists to function in their role as clinical experts. The uneven distribution of specialist nurses over shifts was reported. Sometimes trauma and emergency nurse specialists are allocated to low acuity settings where there is no need for their specialised skills, which also limits their practice role, and thus, the value of their specialised skills is disregarded:

‘On our shift we are three specialised nurses, which is an advantage. But then you get a shift where there’s only one specialised nurse on duty. It takes a lot from a person. So, it [*staff mix*] actually doesn’t correlate every day with regards to the staff-patient ratio.’ (Participant 6, female, trauma and emergency nurse specialist)

‘I just feel like the actual ward that we [*are currently*] in doesn’t even need trauma trained people. If you really want a good trauma trained person, put them in place, so they will be utilised correctly. Ja [*yes*], like at least give them an environment that’s gonna be conducive or would be satisfying to them.’ (Participant 5, female, trauma and emergency nurse specialist)

#### Service demands overwhelm capacity

The high workload in the practice setting impeded specialised skill implementation. A typical night shift in an emergency unit with time pressure and service demands increased the workload to such an extent that they did not have time to utilise their specialised skills (Stevens 2021) as depicted:

‘So, with regards to overcrowding, it gets terrible at times because the last couple of nights we had about 95 patients in our unit. So, it was difficult for us to apply your specialised knowledge, you [*just*] had to do your basic nursing care.’ (Participant 6, female, trauma and emergency nurse specialist)

Frustration was expressed with having to nurse a ‘red’ triaged patient who required immediate intervention. These high-priority patients should be admitted to an appropriate resuscitation bed equipped with electronic devices, such as infusion pumps and cardiac monitors. However, because of overcrowding, an impractical situation was created where there was no access to such equipment to provide immediate life-saving treatment (Stevens 2021):

‘… I must now stabilise him on a chair until I have space for him [*in the resus area*]. And then the doctor [*says:*] “Sister but you only gave the fluid, you didn’t start that Actrapid infusion”. Where must I start that Actrapid infusion? I cannot start it on a chair [*without equipment*] for my patient.’ (Participant 2, female, trauma and emergency nurse specialist)

Trauma and emergency nurse specialists are competent in identifying any deterioration in a patient’s status and implementing appropriate management to prevent serious complications (Stevens 2021). However, overwhelming patient volumes in emergency settings compromise the use of such skills:

‘I mean, the ward can get so full that a patient will lay on a stretcher bed in the passage. So, your patient load becomes ridiculous. Because … Ja [*yes*], then the workload of patients is more so you don’t get out to everything. So it’s harder to pick, not harder to pick up … but it’s harder to get to, if there’s so many patients in a ward. And I mean it’s a surgical ward, so we have from vascular to abdominal, urology and endocrine. So, I mean, if your place is that full, it’s hard to pick up who really needs more attention.’ (Participant 5, female, trauma and emergency nurse specialist)

Non-emergency patients frequently occupy and obstruct bed spaces in trauma and emergency units while awaiting transfer to general wards or intensive care units (ICUs). This situation arises as a result of staff shortages and limited bed availability within the facility itself or because of the unavailability of beds at the designated tertiary referral hospital (Stevens 2021):

‘Because of the shortage of staff … and of space and of beds … you end up nursing general patients that belong in the ward …like the medical patient, surgical patient, gynae patients, and also psychiatry patients become your responsibility. So, you are not doing what you are trained to do as a trauma nurse. And then we are nursing ICU patients in our resus. Because we only have two tertiary hospitals in the Western Cape, so if their ICU’s is full, then we sit with ICU patients for … days.’ (Participant 4, female, trauma and emergency nurse specialist)

#### Limited practice opportunities lead to the loss of specialised skill competencies

The prohibition to utilise specialised skills inhibits the safeguarding of specialised skills in practice environments. Consequently, skills and confidence are lost. A participant referred to ‘shrinking’ in their specialist roles as opposed to expanding their roles with their specialised skills when they are only allowed to receive and carry out orders, instead of acting from a competence base:

‘So, it’s kind of hard, especially if you’re not exposed to things all the time. I mean I’m [*a*] human being; I’m going to forget things if I’m not going to be doing it on a daily basis.’ (Participant 5, female, trauma and emergency nurse specialist)

‘I feel we are slowly but surely shrinking into just [*saying*] yes, no … Ja [*yes*], I will do that now [*taking orders only*]. We never actually take initiative for anything.’ (Participant 3, female, trauma and emergency nurse specialist)

Practices thus vary significantly between services, which on the one end of the continuum are experienced as extreme overload with inadequate resources, and on the other end of the continuum, available trauma and emergency nurse specialists are not used to their full capacity as a result of an inadequate skill mix or not used in their specialty at all, with subsequent valuable skill losses.

### Theme 3: Disregard for trauma and emergency nurse specialists’ role and value of specific skills

The third theme in Table 1 refers to the disregard for the trauma and emergency nurse specialist’s role and value of their skills and has the sub-theme of a lack of awareness, recognition and appreciation of nurse specialised role and skill sets in the multidisciplinary trauma and emergency team.

#### A lack of awareness, recognition and appreciation of specialised role and skill sets in the multidisciplinary team

Participants mention a lack of recognition among junior doctors and those not aware of trauma and emergency nurse specialist’s knowledge and skills, and for the valuable role they play in the multidisciplinary team. This lack of recognition results in having to fight for the right to use their knowledge and skill sets:

‘It goes back to doctors being unaware of our capabilities, and therefore they don’t understand our role, especially as trauma nurses [*trauma and emergency nurse specialists*], because I think they’ve struggled to switch, between knowing what the general nurse [*professional nurse without specialised training*] does and what the nurse specialist does.’ (Participant 7, female, trauma and emergency nurse specialist)

‘In the beginning it was quite a bit of … almost a combative fight [*between*] you and the doctor. Because the doctor feels or that he is mostly [*up*] there and that your knowledge doesn’t matter in any way. Then you get your junior doctors and they feel that they are [*the*] most important. They wouldn’t even acknowledge the fact that you’re there.’ (Participant 6, female, trauma and emergency nurse specialist)

‘But I must say. It’s your comm serve doctors [*newly qualified doctors completing their required community service year post training*] and your interns that think they are all THAT! They treat you with such disrespect.’ (Participant 4, female, trauma and emergency nurse specialist)

This lack of recognition, coupled with overt displays of disrespect, leaves trauma and emergency nurse specialists feeling unappreciated and voiceless. It also undermines inter-professional relations and limits the utilisation of their specialised skills within the practice environment:

‘It’s very important, I think, as a trauma nurse specialist, if we would be able to implement all of these skills and have a voice within the multidisciplinary team. Because the one thing that we are lacking is the voice within the team. No one listens to the nurse and no one values the nurse’s opinion … no one take note, because within the multidisciplinary team, we’re not noticed or recognised.’ (Participant 7, female, trauma and emergency nurse specialist)

A participant shared her idea of fulfilling her potential as a valuable contributor to the multidisciplinary team in their role as trauma and emergency nurse specialists:

‘And then others can draw knowledge from you, [*so*] that [*the*] future can seem different of what a nurse should be doing in a multidisciplinary team. Not just writing and taking orders but being part of the discussion … it will improve the quality of that patient, and it might actually also improve the turnaround [*time*] of patients within the emergency center.’ (Participant 3, female, trauma and emergency nurse specialist)

The inability among the team members to fully recognise trauma and emergency nurse specialists’ contributions in the trauma and emergency room stems from a lack of awareness about their knowledge and skill set and subsequent distrust displayed about their function and abilities.

## Discussion

Uncertainty is experienced by qualified trauma and emergency nurse specialists in this study because of a lack of alignment of SOPs and their job descriptions with the competencies obtained for the qualification of trauma and emergency nursing. Barnard et al.’s (2023:14) South African study confirms that fewer than half of specialised trained trauma and emergency nurses had autonomy to make decisions about patient care in their workplace. This correlates with the report of the trauma and emergency nurse specialists in this study about their inability to engage in practices, which are perceived by other members of the multidisciplinary team as beyond the accepted SOP of a specialist nurse. Well-devised SOPs and guidelines create the space and opportunity for nurses to become leaders of the multidisciplinary resuscitation teams, as demonstrated by Espinosa et al. (2020:1) and Barrett, Webb and Louw (2010:15) in their description of this concept in the use of intravenous fluids and the massive blood transfusion protocol in California. Support from the medical team in identifying practice needs and compiling SOPs and guidelines to guide trauma and emergency nurse specialists in the absence of doctors empowers them. Sa et al. (2023:6560) provide evidence of the accuracy of trauma and emergency nurse specialists to recognise physiological deterioration, contributing to improved patient outcomes. For this reason, Brysiewicz and Bruce (2008:130) and Feringa, De Swardt and Havenga (2018:87) have called for several decades now for a review of an SOP by SANC to include the special skills of trauma and emergency nurse specialists. This would legitimise their skills and empower them to fulfil their capabilities as trauma and emergency nurse specialists.

Setting specific guidelines, compiled and supported by the trauma multi-disciplinary team, will ensure that trauma and emergency nurse specialists are empowered to initiate immediate life-saving protocols in specific situations. Ruiz (2018:40) mentions some dissent about this opinion. Some participants in her United Kingdom (UK) study felt that APN flexibility should be respected to some degree, while others felt safe within the protection that guidelines give them. It seems that the degree to which APNs and the multidisciplinary team work together influences feelings of security for APNs and trust of multidisciplinary team members in them. Dubree et al. (2015:45) confirm that highly trained empowered nurses’ excelling in their clinical practice provide quality healthcare and encourage physicians’ trust in the advanced nurse practitioner’ abilities.

In Ruiz’s (2018:37) study, 82% of the UK multidisciplinary team believed clearer role definitions for the trauma and emergency nurse specialists were required, which echoes the sentiments of an earlier study in Wales (Jones et al. 2015). More recently, Espinosa et al. (2020:1) and Donelan et al. (2020:599) advocate for clear delineations of task expectations and clearly demarcated roles and responsibilities between nurse and physician leaders. This is seen as the cultivation of an organisational structure of collaborative and trusting inter-professional relationships that would support the unique contributions by trauma and emergency nurse specialists to the team and that would capitalise on the full extent of their training to meet the healthcare needs of the country.

Service demands that overwhelm capacity and result in inappropriate allocation of staff without consideration of the necessary skills mix provide the sense of trauma and emergency settings being oppressive. Hardcastle et al. (2016:182) ascribe the inability to cope with service demands in South Africa to the ineffective planning and design of trauma and emergency units, causing blocked referral pathways and limited resuscitation capacities, with the subsequent prioritisation of routine care of long-term patients not needing specialist skills. Furthermore, primary healthcare patients visit trauma and emergency centres by preference, instead of their local clinics, passing on the burden of among others, human immunodeficiency virus (HIV) and tuberculosis (TB) services and non-emergency cases to trauma and emergency nurse specialists at emergency centres. Vogel et al. (2019:2617) confirm the choice of patients to attend trauma and emergency centres for health care, rather than attendance of primary care services. Horvath et al. (2023:11) ask for clear commitment from governments to build a sustainable healthcare system by ensuring that primary healthcare services function adequately to prevent overload on emergency services and admissions to further levels of care.

Further oppressive practices are perceived in this study in the form of inappropriate staffing allocations and inadequately balanced skill mixes that undermine the effective utilisation and facilitate loss of expertise. Lamb et al. (2018:403,407), Wolf et al. (2017) and Jones et al. (2015) propose that supportive nursing leadership ensures the utilisation of advanced nursing practice in emergency care settings. Such supportive leadership is needed in ensuring adequate staffing levels for a fair patient-nurse ratio and equal distribution of speciality skills in the skill mix of the different shifts. McConnell, Slevin and McIlfatrick (2013:81) mention from a UK perspective that diversion of emergency nurse practitioners during periods of high staff absenteeism and increased service demands restricts opportunities for skill implementation. Nakweenda, Anthonie and Van der Heever (2022:1) report on various staffing strategies followed by management to deal with the critical shortage of specialist nurses in the intensive care units of Namibia, but that are still unable to address inadequate staff skill mix and increased workloads, similar to more affluent countries such as reported by Haryanto (2019) about the United States of America.

In sub-Sahara Africa, countries are resource poor and have funding challenges to ensure sufficient training of nurses in general and specifically specialist nurses (Oleribe et al. 2019), which means that countries on this continent struggle to create and fill the necessary posts in emergency and trauma units.

A disregard for the trauma and emergency nurse specialist role and their valuable specialised skills and expertise existed in the multidisciplinary team. As a result, they were unable to apply their skills, which caused feelings of disempowerment. Wolf et al. (2017:432) and Lamb et al. (2018:408) concur that the implementation of specialised skills is facilitated by respect for advanced knowledge and expertise in skills in the provision of high-quality advanced-level nursing care. Display of such respect supports the participants’ self-worth and credibility of their specialised role, which sets the stage for nurse-physician collaboration in a supportive organisational culture to promote advanced nursing practice (Wolf et al. 2017:431) (Stevens 2021). Ruiz (2018:38) reports a weakening resistance from doctors and other multi-disciplinary team members to trauma and emergency nurse specialist practice in the UK, which should be a goal in South Africa, where the need is even higher for trauma and emergency nurse specialist care in trauma and emergency departments.

### Recommendations

In South Africa as an LMIC, the inappropriate use of trauma and emergency nurse specialists can be considered as wasteful expenditure and needs to be rectified by addressing these identified barriers voiced by this important stakeholder group in South African healthcare. To be able to fulfil recognised trauma and emergency nurse specialists’ roles optimally in caring for an LMIC’s varied population needs, the uncertainty regarding legal accountability, autonomy and specialised practice responsibility caused by inhibitory governmental legislation from the Department of Health, a lack of specific professional regulations from SANC and institutional guidelines from specific hospitals need to be addressed. Inclusion by the SANC of all stakeholders in the development of a specific regulation for trauma and emergency nurse specialists should be encouraged, which needs to be legislated by the Minister of Health. At workplaces, the specialised skills of these nurses should be protected by engagement with management and the multidisciplinary team in negotiation for and recognition of extended job descriptions. Together with continuous updating of knowledge with the help of short courses and medical practitioner discussions, verifiable proficiency for practising, refining and building confidence in their specialist skills will be ensured. Ultimately, this will promote productivity, safe staffing levels and quality of care. Recognition of trauma and emergency nurse specialists as part of the trauma and emergency team by way of interprofessional role awareness, hospital management support and involvement in decision-making processes on all government levels is thus paramount.

### Limitations

The COVID-19 pandemic lockdown provided limitations to this study when some trauma and emergency nurse specialists were overwhelmed and not able to respond to requests for interviews because of workloads and family responsibilities. Furthermore, the lack of data about the topic in South Africa to compare with the international context posed a limitation. Lastly, the positionality of the researcher as an educator of the trauma and emergency specialist course could have introduced some bias via the participants’ reactions. To counteract this, the researcher provided a thorough explanation about the need and value of their perspectives to address barriers that trauma and emergency specialist nurses experience. In addition, bracketing of her own ideas about the position of the trauma and emergency nurse specialists was further applied by the researcher to ensure that the perspectives of the participants were accurately accounted for.

## Conclusion

This research provided an understanding of the application of specialised skills by trauma and emergency nurse specialists. The findings suggest that trauma and emergency nurse specialists experience barriers to the optimal use of their specialised skill set. These barriers include uncertainty about their legal accountability and specialised practice responsibility as a result of SOP limitations and non-alignment of job descriptions with newly acquired specialised skills. Further barriers are oppressive practice environments where service demands overwhelm capacity, not being allowed to use their specialised skills and limited practice opportunities, which eventually leads to loss of specialised competencies. Lastly, there is a disregard for their specialist role, shown by inappropriate staff allocations and skill mixes, and a lack of recognition, awareness and valuing of their specialised role in the multidisciplinary trauma and emergency team.

## References

[CIT0001] Achoki, T., Sartorius, B., Watkins, D., Glenn, S.D., Kengne, A.P., Oni, T. et al., 2022, ‘Health trends, inequalities and opportunities in South Africa’s provinces, 1991–2019: Findings from the global burden of disease 2019 study’, *Journal of Epidemiology and Community Health* 76(5), 471–481. 10.1136/jech-2021-217480 35046100 PMC8995905

[CIT0002] Barnard, N., Rothmann, S., De Beer, L.T. & Lubbe, W., 2023, ‘Burnout of emergency nurses in a South African context: The role of job demands and resources, and capabilities’, *Frontiers in Psychology* 14, 1119063. 10.3389/fpsyg.2023.1119063 37275737 PMC10233105

[CIT0003] Barrett, C., Webb, M. & Louw, V., 2010, ‘The role of nurses in massive transfusion’, *Professional Nursing Today* 14(3), 12–15, viewed 12 December 2024, from https://www.researchgate.net/publication/254864082_The_role_of_nurses_in_massive_transfusion/link/0deec51ff70017b051000000/download.

[CIT0004] Braun, V. & Clarke, V., 2020, ‘One size fits all? What counts as quality practice in (reflexive) thematic analysis?’, *Qualitative Research in Psychology* 18(3), 328–352. 10.1080/14780887.2020.1769238

[CIT0005] Brysiewicz, P. & Bruce, J., 2008, ‘Emergency nursing in South Africa’, *International Emergency Nursing* 16(2), 127–131. 10.1016/j.ienj.2008.01.001 18519064

[CIT0006] Crawford, C., 2019, ‘Addition of advanced practice registered nurses to the trauma team: An integrative systematic review of literature’, *Journal of Trauma Nursing* 26(3), 141–146. 10.1097/JTN.0000000000000439 31483771

[CIT0007] Department of Health, 2015, *White paper on national health insurance*, Government Gazette, Pretoria.

[CIT0008] Donelan, K., DesRoches, C.M., Guzikowski, S., Dittus, R.S. & Buerhaus, P., 2020, ‘Physician and nurse practitioner roles in emergency, trauma, critical, and intensive care’, *Nursing Outlook* 68(5), 591–600. 10.1016/j.outlook.2020.04.010 32622648 PMC7241342

[CIT0009] Dubree, M., Jones, P., Kapu, A. & Parmley, C.L., 2015, ‘APRN practice: Challenges, empowerment, and outcomes’, *Nurse Leader* 13(2), 43–49. 10.1016/j.mnl.2015.01.007

[CIT0010] Espinosa, E., Gallagher, B., Kosak, D., Maury, J., Pedicini, D., Potter-Fortney, A. et al., 2019, *Role of the trauma nurse in massive transfusion protocol*, viewed 12 December 2024, from https://www.traumanurses.org/_resources/documents/events/conference/2019/posters/E104.pdf.

[CIT0011] Feringa, M.M., De Swardt, H.C. & Havenga, Y., 2018, ‘Registered nurses’ knowledge, attitude, practice and regulation regarding their scope of practice: A literature review’, *International Journal of Africa Nursing Sciences* 8, 87–97. 10.1016/j.ijans.2018.04.001

[CIT0012] Hardcastle, T.C., Clarke, D., Oosthuizen, G. & Lutge, E., 2016, ‘Trauma, a preventable burden of disease in South Africa: Review of the evidence, with a focus on KwaZulu-Natal’, *South African Health Review* 1, 179–189, viewed 12 December 2024, from http://journals.co.za/content/healthr/2016/1/EJC189309.

[CIT0013] Haryanto, M., 2019, ‘Nursing shortage: Myth or fact?’, *Orthopaedic Nursing* 1(1), 1–2. 10.1097/NOR.0000000000000535 30676566

[CIT0014] Horvath, S., Visekruna, S., Kilpatrick, K., McCallum, M. & Carter, N., 2023, ‘Models of care with advanced practice nurses in the emergency department: A scoping review’, *International Journal of Nursing Studies* 148, 1–14. 10.1016/j.ijnurstu.2023.104608 37801938

[CIT0015] Jones, A., Powell, T., Watkins, D. & Kelly, D., 2015, ‘Realising their potential? Exploring interprofessional perceptions and potential of the advanced practitioner role: A qualitative analysis’, *BMJ Open* 5(12), 1–8. 10.1136/bmjopen-2015-009740 PMC4679915 26656024

[CIT0016] Kandrack, R., Barnes, H. & Martsolf, G.R., 2019, ‘Nurse practitioner scope of practice regulations and nurse practitioner supply’, *Medical Care Research and Review* 78(3), 208–217. 10.1177/1077558719888424 31729899

[CIT0017] Lamb, A., Martin-Misener, R., Bryant-Lukosius, D. & Latimer, M., 2018, ‘Describing the leadership capabilities of advanced practice nurses using a qualitative descriptive study’, *Nursing Open* 5(3), 400–413. 10.1002/nop2.150 30191074 PMC6121481

[CIT0018] Lecky, F.L., Reynolds, T., Otesile, O., Hollis, S., Turner, H.J., Fuller, G. et al., 2020, ‘Harnessing inter-disciplinary collaboration to improve emergency care in low- and middle-income countries (LMIC): Results of research prioritisation setting exercise’, *BMC Emergency Medicine* 20(68), 1–10. 10.1186/s12873-020-00362-7 32867675 PMC7457362

[CIT0019] Lincoln, Y.S. & Guba, E.G., 1985, *Naturalistic inquiry*, Sage, Beverly Hills, CA.

[CIT0020] Liu, Y., Han, D., Liu, X., Luo, L., Zhou, Q. & Chen, M., 2024, ‘“Trauma nursing practitioners” preferences for training for trauma specialist nurses based on job competencies’, *International Emergency Nursing* 7, 101525. 10.1016/j.ienj.2024.101525 39388824

[CIT0021] McConnell, D., Slevin, O.D. & McIlfatrick, S.J., 2013, ‘Emergency nurse practitioners’ perceptions of their role and scope of practice: Is it advanced practice?’, *International Emergency Nursing* 21(2), 76–83. 10.1016/j.ienj.2012.03.004 23615513

[CIT0022] Nakweenda, M., Van der Heever, A. & Anthonie, M., 2022, ‘Staff shortages in critical care units: Critical care nurses’ experiences’, *International Journal of Africa Nursing Sciences* 17, 100412. 10.1016/j.ijans.2022.100412

[CIT0023] Oleribe, O.O., Momoh, J., Uzohukwu, B.S.C., Mbofana, F., Adebiyi, A., Barbera, T. et al., 2019, ‘Identifying key challenges facing healthcare systems in Africa and potential solutions’, *International Journal of General Medicine* 12, 395–403. 10.2147/IJGM.S223882 31819592 PMC6844097

[CIT0024] Ruiz, L.M., 2018, ‘Multidisciplinary team attitudes to an advanced nurse practitioner service in an emergency department’, *Emergency Nurse* 28(1), 33–42. 10.7748/en.2018.e1793 30277346

[CIT0025] Sa, W., Shuihong, C., Jingfen, J., Mao, Z., Zhiting, G., Danping, Y. et al., 2023, ‘The effect of trauma advanced practice nurse programme at a Level I regional trauma centre in mainland China’, *Nursing Open* 10(9), 6559–6565. 10.1002/nop2.1911 37332249 PMC10416056

[CIT0026] South African Nursing Council (SANC), 2022, *Regulations regarding the scope of practice for nurses and midwifes R2127*, viewed 18 December 2024, from https://www.sanc.co.za/wp-content/uploads/2023/03/Regulations-Scope-of-Practice-Nurse-and-Midwife.pdf.

[CIT0027] Stevens, H., 2021, ‘Trauma and emergency nurse specialists’ perceptions of factors that hinder and facilitate the implementation of specialised skills in their practice within the public health sector in the Western Cape Province’, Masters thesis, Faculty of Medicine and Health Sciences, Stellenbosch University.

[CIT0028] Torrens, C., Campbell, P., Hoskins, G., Strachan, H., Wells, M., Cunningham, M. et al., 2020, ‘Barriers and facilitators to the implementation of the advanced nurse practitioner role in primary care settings: A scoping review’, *International Journal of Nursing Studies* 104, 103443. 10.1016/j.ijnurstu.2019.103443 32120089

[CIT0029] Vogel, J.A., Rising, K.L., Jones, J., Bowden, M.L., Ginde, A.A. & Havranek, E.P., 2019, ‘Reasons patients choose the emergency department over primary care: A qualitative metasynthesis’, *Journal of General Internal Medicine* 34(11), 2610–2619. 10.1007/s11606-019-05128-x 31428988 PMC6848423

[CIT0030] Western Cape Department of Health (WCDoH), 2019, *Annual report 2018–2019*, viewed 18 December 2024, from https://www.westerncape.gov.za/assets/departments/health/annual_report_2019.pdf.

[CIT0031] Wolf, L., Delao, A.M., Perhats, C., Moon, M.D. & Carman, M.J., 2017, ‘The experience of advanced practice nurses in US emergency care settings’, *Journal of Emergency Nursing* 43(5), 426–434. 10.1016/j.jen.2017.04.007 28579285

[CIT0032] World Health Organization (WHO), 2020, *A regional guide to the development of nursing specialist practice*, viewed 18 April 2025, from https://applications.emro.who.int/docs/WHOEMNUR432E-eng.pdf.

[CIT0033] Zaidi, A.A., Dixon, J., Lupez, K., De Vries, S., Wallis, L.A., Ginde, A.A. et al., 2019, ‘The burden of trauma at a district hospital in the Western Cape Province of South Africa’, *African Journal of Emergency Medicine* 9(suppl 1), S14–S20. 10.1016/j.afjem.2019.01.007 31073509 PMC6497867

