# Corrigendum: Barriers to implementing advanced skills in trauma and emergency nurse specialist practice

**DOI:** 10.4102/hsag.v30i0.3228

**Published:** 2025-09-01

**Authors:** Haroldene Stevens, Cornelle Young, Tshepo L. Motsepe, Tendani Mabuda

**Affiliations:** 1 Department of Nursing and Midwifery, Faculty of Medicine and Health Sciences, Stellenbosch University, Cape Town, South Africa

In the published article, Barriers to implementing advanced skills in trauma and emergency nurse specialist practice, there was an error regarding the affiliation(s) for Haroldene Stevens.

Instead of:

Department of Health and Wellness, Western Cape College of Nursing, South Cape Karoo Nursing Campus, George, South Africa.

It should be:

Department of Nursing and Midwifery, Faculty of Medicine and Health Sciences, Stellenbosch University, Cape Town, South Africa.

The authors apologise for this error. The correction does not change the study’s findings, its significance or overall interpretation of its results or the scientific conclusions of the article in any way.

